# Cell-specific transcriptome changes in the hypothalamic arcuate nucleus in a mouse deoxycorticosterone acetate-salt model of hypertension

**DOI:** 10.3389/fncel.2023.1207350

**Published:** 2023-05-24

**Authors:** Valerie A. Wagner, Guorui Deng, Kristin E. Claflin, McKenzie L. Ritter, Huxing Cui, Pablo Nakagawa, Curt D. Sigmund, Lisa L. Morselli, Justin L. Grobe, Anne E. Kwitek

**Affiliations:** ^1^Department of Physiology, Medical College of Wisconsin, Milwaukee, WI, United States; ^2^Genetics Graduate Program, University of Iowa, Iowa City, IA, United States; ^3^Department of Neuroscience and Pharmacology, University of Iowa, Iowa City, IA, United States; ^4^Obesity Research and Education Initiative, University of Iowa, Iowa City, IA, United States; ^5^Fraternal Order of Eagles Diabetes Research Center, University of Iowa, Iowa City, IA, United States; ^6^Cardiovascular Center, Medical College of Wisconsin, Milwaukee, WI, United States; ^7^Neuroscience Research Center, Medical College of Wisconsin, Milwaukee, WI, United States; ^8^Department of Medicine, Division of Endocrinology and Molecular Medicine, Medical College of Wisconsin, Milwaukee, WI, United States; ^9^Comprehensive Rodent Metabolic Phenotyping Core, Medical College of Wisconsin, Milwaukee, WI, United States; ^10^Department of Biomedical Engineering, Medical College of Wisconsin, Milwaukee, WI, United States; ^11^Linda T. and John A. Mellowes Center for Genomic Sciences and Precision Medicine, Medical College of Wisconsin, Milwaukee, WI, United States

**Keywords:** snRNAseq, DOCA-salt, arcuate nucleus, AgRP neurons, microglia, mouse

## Abstract

A common preclinical model of hypertension characterized by low circulating renin is the “deoxycorticosterone acetate (DOCA)-salt” model, which influences blood pressure and metabolism through mechanisms involving the angiotensin II type 1 receptor (AT_1_R) in the brain. More specifically, AT_1_R within Agouti-related peptide (AgRP) neurons of the arcuate nucleus of the hypothalamus (ARC) has been implicated in selected effects of DOCA-salt. In addition, microglia have been implicated in the cerebrovascular effects of DOCA-salt and angiotensin II. To characterize DOCA-salt effects upon the transcriptomes of individual cell types within the ARC, we used single-nucleus RNA sequencing (snRNAseq) to examine this region from male C57BL/6J mice that underwent sham or DOCA-salt treatment. Thirty-two unique primary cell type clusters were identified. Sub-clustering of neuropeptide-related clusters resulted in identification of three distinct AgRP subclusters. DOCA-salt treatment caused subtype-specific changes in gene expression patterns associated with AT_1_R and G protein signaling, neurotransmitter uptake, synapse functions, and hormone secretion. In addition, two primary cell type clusters were identified as resting versus activated microglia, and multiple distinct subtypes of activated microglia were suggested by sub-cluster analysis. While DOCA-salt had no overall effect on total microglial density within the ARC, DOCA-salt appeared to cause a redistribution of the relative abundance of activated microglia subtypes. These data provide novel insights into cell-specific molecular changes occurring within the ARC during DOCA-salt treatment, and prompt increased investigation of the physiological and pathophysiological significance of distinct subtypes of neuronal and glial cell types.

## Introduction

In the 1970’s, (Laragh, 1971) established that approximately 25% of humans with essential hypertension exhibit low plasma renin activity. More recent studies have demonstrated that low-renin hypertension is more common among the elderly, patients of African ancestry, those with heart or renal failure, and women with preeclampsia (Shah, 2006; Buffolo et al., 2020; Joseph et al., 2021). Increased understanding of the causes and consequences of these low-renin forms of hypertension is necessary to further the development of new efficacious, safe, and personalized therapeutic approaches.

One common model of low-renin hypertension involves delivery of deoxycorticosterone acetate (DOCA) and a high dietary sodium load. This “DOCA-salt” model resulted in robust hypertension, proteinuria, and damage to various cardiovascular organs (Selye et al., 1943). Substantial evidence supports a major role for the central nervous system in the pathogenesis of DOCA-salt hypertension (Schenk and Mcneill, 1992; Yemane et al., 2010). For example, activation of the renin-angiotensin system (RAS) in the brain is required for both development and maintenance of DOCA-salt hypertension as these effects are attenuated by brain-specific delivery of angiotensin converting enzyme inhibitors or angiotensin II type 1 receptor (AT_1_R) antagonists (Itaya et al., 1986; Kubo et al., 2000; Park and Leenen, 2001). A role for microglia in the cerebrovascular effects of DOCA-salt treatment has also been previously identified (Koizumi et al., 2019). Thus, the cardiovascular effects of DOCA-salt appear to involve effects mediated through multiple distinct cell types within the brain.

Several studies have demonstrated that DOCA-salt treatment affects energy homeostasis through the stimulation of resting metabolic rate (RMR) (Goodwin et al., 1969; Gavras et al., 1975; Campión et al., 1998; Grobe et al., 2010). We and others have implicated the RAS and AT_1_R signaling within the arcuate nucleus of the hypothalamus (ARC) in RMR responses to DOCA-salt. The Brooks group and others have also demonstrated a role for this brain region (and ANG action therein) in blood pressure control (Grobe et al., 2010, 2011; Claflin et al., 2017; Shi et al., 2017, 2020, 2021; Jiang et al., 2020; Mehay et al., 2021; Oliveira et al., 2021). These findings prompt further investigation of the effects of DOCA-salt treatment upon the ARC, to better understand the interplay between blood pressure and metabolic control mechanisms within this region.

To characterize the cell-specific changes that occur within the ARC following DOCA-salt treatment, we used single-nucleus RNA sequencing (snRNAseq) to individually interrogate the transcriptomes of the many cell types within the ARC in mice treated with DOCA-salt or sham treatments. The resulting dataset provides novel insight into cell-specific molecular changes that occur within the ARC in response to DOCA-salt, which may represent novel contributors to or consequences of hypertension and hypermetabolism occurring in this model of low-renin hypertension.

## Materials and methods

### Animals

All studies were approved by the University of Iowa and Medical College of Wisconsin Institutional Animal Care and Use Committees, and conform to expectations laid out in the Guide for the Care and Use of Laboratory Animals, 8th Edition (National Research Council, 2011). Wildtype male C57BL/6J mice were obtained from the Jackson Laboratories (catalog #000664) at 5–6 weeks-of-age. All mice were housed at 24 ± 2^°^C with a 12:12 light-dark cycle in an AAALAC-approved animal facility at the University of Iowa (Iowa City, IA). Animal protocols were approved by the Institutional Animal Care and Use Committee at the University of Iowa.

### Study protocol and snRNAseq

After weaning and until shipping, animals at Jackson Laboratories were maintained on LabDiet JL Rat and Mouse/Auto 6F 5K52 diet (22% kcal from protein, 16% kcal from fat, and 62% kcal from carbohydrates; and providing 0.26% Na). Upon arrival at the University of Iowa, mice were immediately switched to Teklad 7913 diet (23% kcal from protein, 18% kcal from fat, 59% kcal from carbohydrate; and providing 0.3% Na) for the remainder of the study. Mice were weighed weekly and body composition was assessed at seven and 10 weeks-of-age using time-domain nuclear magnetic resonance (NMR; Bruker, Billerica, MA, United States, model LF50). At 7 weeks-of-age, animals were randomly assigned to either sham surgery or surgical implantation of a 50 mg pellet of deoxycorticosterone acetate (DOCA; Sigma-Aldrich, St. Louis, MO, United States) into the subcutaneous space below the scapulae under isoflurane anesthesia. Before surgery, animals were matched for body mass and body composition. Mice were supplied water *ad libitum*, and DOCA-salt mice were provided *ad libitum* access to a 0.15 M NaCl drink. We have previously documented that this intervention causes moderate hypertension, increased RMR, elevated arginine vasopressin secretion, and hypernatremia (Grobe et al., 2010, 2011; Hilzendeger et al., 2013; Claflin et al., 2017; Sandgren et al., 2018; Patil et al., 2022). At 10 weeks-of-age, mice were euthanized by CO_2_ asphyxiation, trunk blood was collected into lithium-heparin-coated tubes, and brains were immediately frozen in isopentane over dry ice. Plasma electrolytes, blood-urea-nitrogen (BUN), and hematocrit were determined using an iSTAT handheld blood analyzer (iSTAT, Chem8 + cartridges) (Patil et al., 2022). Bilateral arcuate punches from 10 μm-thick coronal slices were obtained from the remaining brains using a brain punch kit (0.74 mm in diameter) from Stoelting Co. (Illinois, USA) and stored in RNALater ICE (Thermo-Fischer, Waltham, MA, United States) at −80^°^C until day of extraction.

Four mice per condition were selected for sequencing. Post-hoc comparisons of various physiological endpoints confirm that the remaining four animals (two pools within each group) were indeed representative of the original cohort groups. For example, relative to the *n* = 4 sequenced Sham animals, the *n* = 4 sequenced DOCA-salt animals exhibited hypernatremia (+9 mEq/L, *p* = 0.03), hypokalemia (−4 mEq/L, *p* < 0.01), suppressed BUN (−16 mg/dL, *p* < 0.01), increased hematocrit (+6%, *p* = 0.03), increased kidney mass (+70 mg, *p* = 0.02), and reduced adrenal mass (−3 mg, *p* = 0.02). The *n* = 3–4 animals per group that were not used for sequencing each failed quality control during nuclei isolation and were therefore excluded.

### snRNAseq and analysis

Tissue was homogenized and nuclei resuspended following previously published methods (Deng et al., 2020). Briefly, bilateral ARC brain punches were homogenized in Nuclei EZ lysis buffer (Sigma-Aldrich, St. Louis, MO, United States). Homogenates were then filtered, collected, and centrifuged in Nuclei EZ lysis buffer. After removal of supernatant, pellets were resuspended in Nuclei Wash and Resuspension buffer. After tissue homogenization and nuclei isolation, samples from individual mice were pooled together according to DOCA-salt treatment pooled to provide sufficient nuclei counts per replicate, resulting in *n* = 2 Sham and *n* = 2 DOCA-salt independent biological replicates (Supplementary Table 1). Sequencing libraries were constructed using the Chromium Single Cell3’ GEM, Library & Gel Bead Kit v3 (10X Genomics, Pleasanton, CA, United States) and sequenced on an Illumina HiSeq 4000 with 150 base pair (bp) paired-end reads (Iowa Institute of Human Genetics, University of Iowa). FASTQ sequencing files (publicly available: GSE221367) were aligned to the mouse reference genome (GRCm38/mm10) and feature-barcode matrices were generated with Cell Ranger v3.0.1 (10X Genomics, Pleasanton, CA, United States). Dataset quality was confirmed by comparing the current dataset to our previously published snRNAseq datasets (Supplementary Table 2; Deng et al., 2020).

Further analyses were carried out using the R package Seurat v4.0.4. Low-quality nuclei (samples) from the four datasets were filtered out as follows: a sample must express more than 200 genes but less than 7,000 (min.features = 200, nFeature < 7,000); a sample’s gene expression profile must be composed of less than 15% mitochondrial genes (percent.mt < 15); a sample must have less than 25,000 total transcripts detected (nCount_RNA < 25,000) (Supplementary Figures 1, 2). Additionally, lowly expressed genes (expressed in less than five samples) were excluded genes (min.cells < 5). Datasets continued through preparations for integration by log normalizing gene expression, cell cycle scoring, and SC transformation (SCTransform). Datasets were integrated using 2,000 anchors and 1:25 principal component analysis (PCA) dimensions. FindNeighbors was used to compute the k.param (20) nearest neighbors for the integrated dataset (PCA dimensions = 1:30). A shared nearest neighbor modularity optimization based clustering algorithm was used in FindClusters to identify clusters of nuclei (resolution = 1.3). Cell type markers were defined by comparing each cluster to all other clusters (FindAllMarkers, min.pct = 0.25). Sub-clustering of C25_Microglia 2 and clusters with neuropeptide-related markers (Clusters 12, 16, 17, 19, 22) was conducted following similar steps as the initial clustering. Briefly, subsets of the full dataset were created, data was rescaled, PCA was rerun, k nearest neighbors was redone (FindNeighbors PCA dimensions for microglia = 1:30; PCA dimensions for neuropeptide = 1:25), and clusters were identified using FindClusters (microglia resolution = 0.5, neuropeptide resolution = 0.6). Cell types were assigned to clusters using four previous studies reporting single-cell transcriptomics within the mouse ARC and other sources (Ciofi et al., 1994; Holmstrand et al., 2014; Jeong et al., 2016; Campbell et al., 2017; Romanov et al., 2017; Han et al., 2018; Moore et al., 2018; Deng et al., 2020; Brunet Avalos and Sprecher, 2021); if no previous data were found clusters were named using the top two uniquely expressed genes.

### Statistical analysis

Physiological effects of DOCA-salt treatment on body mass, body composition, and blood analytes were assessed using two-way ANOVA with Šídák multiple comparison procedure or two-tailed independent *t*-test. The percentage of Sham and DOCA-salt nuclei within both microglia clusters and in individual microglia clusters was compared to the dataset contribution percentages (Sham = 25%, DOCA = 75%) using the Chi-square test in GraphPad Prism 9.5.1.

Differentially expressed genes were determined in Seurat using the following: FindMarkers (test.use = “MAST”, logfc.threshold = 0.1, min.pct = 0.25) (Finak et al., 2015). Inter-cluster differentially expressed genes (cluster vs. cluster not considering Sham or DOCA-salt) were filtered further for a minimum average fold change = 1.19 and a false discovery rate <5%. Intra-cluster differentially expressed genes (Sham vs. DOCA-salt within one cluster) were calculated only in clusters with at least 10% sample contribution from either Sham or DOCA-salt datasets and were filtered further for a minimum fold change = 1.07 and a false discovery rate <5%. Note that the neuropeptide-related clusters underwent DEG analysis after subclustering, separately from the primary clusters. Please note that in tables displaying DOCA-salt treatment differentially expressed genes, “pct.1” refers to the percent of nuclei within a cluster that expresses a given gene in the DOCA-salt group and “pct.2” refers to the percent of nuclei within a cluster that expresses a given gene in the Sham group.

## Results

### Physiological effects of DOCA-salt

**FIGURE 1:**
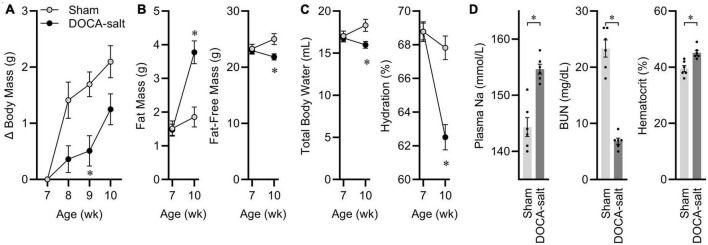
Deoxycorticosterone acetate (DOCA)-salt caused expected physiological effects. **(A)** Body mass gains. Age *p* < 0.01, DOCA-salt *p* < 0.01, Age × DOCA-salt *p* < 0.01. **(B)** Body composition. Fat: Age *p* < 0.01, DOCA-salt *p* = 0.02, Age x DOCA-salt *p* < 0.01. Fat-free mass: Age *p* = 0.26, DOCA-salt *p* = 0.10, Age × DOCA-salt *p* < 0.01. **(C)** Hydration status. Total body water: Age *p* = 0.26, DOCA-salt *p* = 0.10, Age × DOCA-salt *p* < 0.01. Hydration: Age *p* < 0.01, DOCA-salt *p* < 0.01, Age × DOCA-salt *p* < 0.01. **(D)** Plasma sodium (Na), blood urea nitrogen (BUN), and hematocrit. For all panels, *n* = 7 Sham, eight DOCA-salt male C57BL/6J. Data are presented as mean ± SEM and analyzed by two-way ANOVA and Šídák multiple comparison procedure or two-tailed independent *t*-test; *p < 0.05.

At 7 weeks-of-age, eight male C57BL/6J mice started DOCA-salt treatment, while seven littermates underwent sham treatment. Before surgery, animals were matched for body mass and body composition. 3 weeks after surgery, mice treated with DOCA-salt exhibited a suppressed growth trajectory (Figure 1A), and this was the result of reduced fat-free mass, despite an expansion of fat mass (Figure 1B). After 3 weeks of treatment, total body water [estimated as 73.2% of fat-free mass (Segar et al., 2020)] was therefore suppressed by DOCA-salt, resulting in a significant reduction in total body hydration (Figure 1C). Further confirming the efficacy of this DOCA-salt paradigm to alter fluid and electrolyte status after 3 weeks of treatment, plasma Na was significantly increased, blood urea nitrogen was significantly reduced, and hematocrit was significantly increased (Figure 1D). Finally, tissue masses were assessed, and consistent with previous reports of this paradigm in C57BL/6J mice maintained on Teklad 7013, DOCA-salt caused increased renal mass and reduced adrenal mass but no consistent effect on cardiac mass (Supplementary Table 3). From this cohort of animals, two pairs of mice were used as representatives to be processed for snRNAseq analysis of ARC transcriptomes.

### snRNAseq dataset generation

**FIGURE 2:**
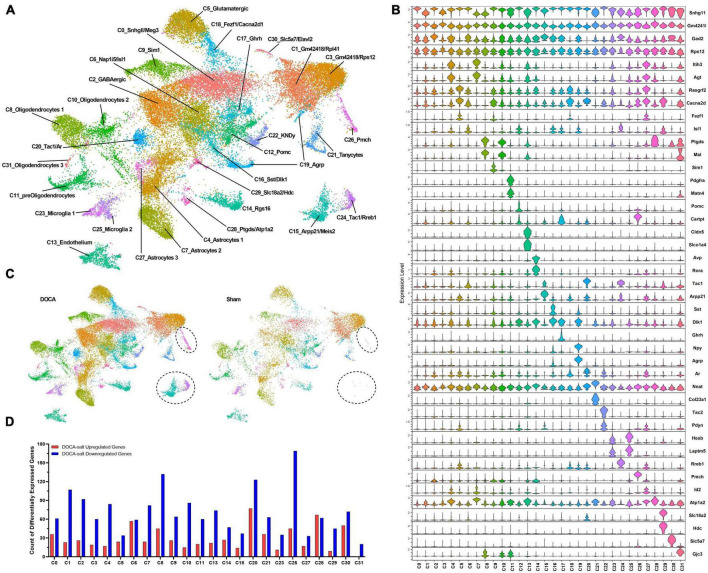
Deoxycorticosterone acetate (DOCA)-salt treatment induces differential expression of genes in individual cell types of the arcuate nucleus of the hypothalamus (ARC). **(A)** Uniform manifold approximation and projection (UMAP) plot of nuclei clusters identified by Seurat. **(B)** Expression of top marker genes that uniquely identify clusters. **(C)** UMAP plot of nuclei clusters, split by condition (Sham, DOCA-salt) with circles highlighting missing C15, C24, C26 clusters within the Sham dataset. **(D)** Differential gene expression counts by cluster between DOCA-salt and Sham conditions in all analyzed clusters (C15, C24, and C26 not analyzed due to poor representation from Sham datasets) except neuropeptide-related clusters (C12, C16, C17, C19, and C22).

At 10 weeks-of-age, nuclei from the ARC were isolated following published methods and used for snRNAseq (Supplementary Table 2; Deng et al., 2020). Quality control measures in the current dataset were comparable to a previous published snRNAseq study from this group (Supplementary Table 3). The R package Seurat 4.1.0 was used to perform filtering of poor-quality nuclei and unbiased clustering (Supplementary Figures 1, 2), identifying 32 clusters (Figure 2A; Hao et al., 2021). Clusters were characterized by the top uniquely-expressed genes in each cluster (Figure 2B and Supplementary Table 4). Most clusters contained nuclei from both Sham and DOCA-salt conditions, however, C15, C24, and C26 were dominated by DOCA-salt nuclei (Sham nuclei: C15 = 0.4%, C24 = 0.5%, C26 = 4.0%) (Figure 2C and Supplementary Table 5). Multiple clusters with neuropeptide-related markers (C12, C16, C17, C19, and C22) and C25 were subclustered using the same algorithms to better classify subtypes of AgRP neurons and activated microglia, respectively, identifying 12 unique neuropeptide-related subclusters (N_SC) (Supplementary Table 6) and five unique activated microglia subclusters (M_SC) (Supplementary Table 7).

### Differential gene expression and gene enrichment analyses

Clusters were selected for differential gene expression (DEG) analysis in Seurat using MAST (Finak et al., 2015) if the clusters were composed of a minimum of 10% of a given condition, to ensure reliable results. Therefore, clusters C15, C24, and C26 that were dominated by DOCA-salt nuclei (<5% Sham nuclei) were not analyzed. Interestingly, DOCA-salt appeared to decrease gene expression more often in the inter-cluster DEG analysis, with 23 out of the 24 analyzed clusters having a greater count of downregulated genes than upregulated genes (Figure 2D). All DEGs were submitted to ShinyGO 0.75 for gene enrichment analysis using Gene Ontology Biological Processes (GOBP), GO Molecular Function (GOMF), KEGG, Biocarta, and Reactome (Ge et al., 2020). Fold enrichment (FE) values are included in the text for all highlighted enrichment terms.

### Multiple subtypes of AgRP neurons

**FIGURE 3:**
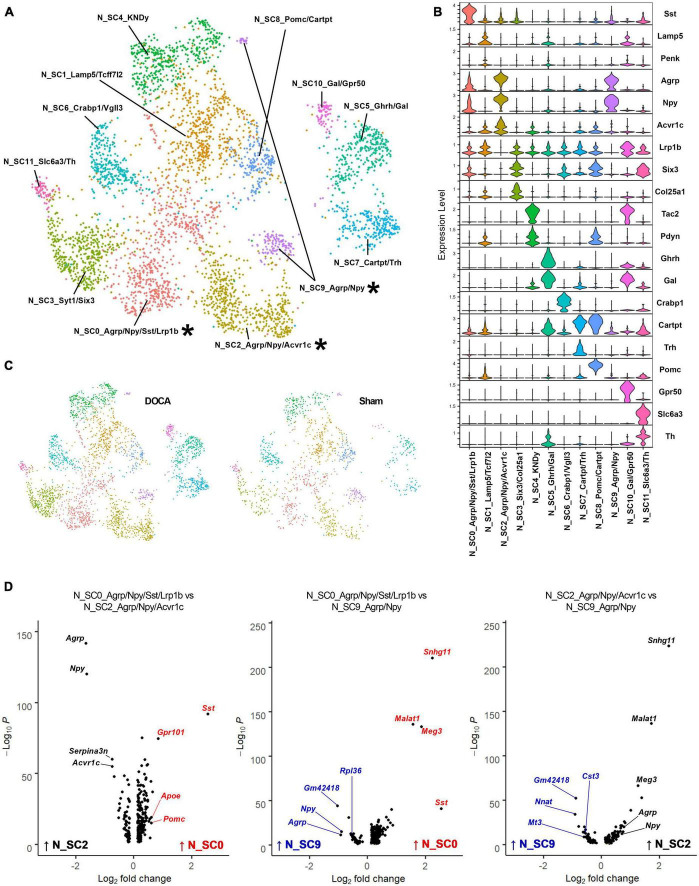
Sub-clustering of neuropeptide-related cell types reveals three subtypes of Agouti-related peptide (AgRP) neurons. **(A)** Uniform manifold approximation and projection (UMAP) plot of neuropeptide-related subclusters identified by Seurat with AgRP subtypes noted with an asterisk (*). **(B)** Expression of top marker genes that uniquely identify subclusters. **(C)** UMAP plot of nuclei sub-clusters, split by condition [Sham, deoxycorticosterone acetate (DOCA)-salt]. **(D)** Volcano plots of differentially expressed genes between pairs of the three AgRP neuronal subtypes N_SC0_Agrp/Npy/Sst/Lrp1b (red), N_SC2_Agrp/Npy/Acvr1c (black), and N_SC9_Agrp/Npy (blue).

Previous single-cell and single-nucleus RNAseq studies have identified numerous neuronal subtypes with high expression of various neuropeptides (Campbell et al., 2017; Romanov et al., 2017), and our previous work has demonstrated a major role for AT_1_R signaling specifically within AgRP neurons for the integrative control of energy expenditure (Claflin et al., 2017; Morselli et al., 2018). Our initial clustering analysis identified only a handful of known neuron subtypes. To determine if our dataset contained other neuropeptide-related neuronal subtypes, clusters C12_Pomc, C16_Sst/Dlk1, C17_Ghrh, C19_Agrp, and C22_KNDy with neuropeptide marker genes were subclustered. Twelve subclusters of neuropeptide-related neurons were identified with good distribution of Sham and DOCA-salt nuclei in each subcluster (Figures 3A, B and Supplementary Tables 6, 8), which included three clusters expressing *Agrp* and *Npy* (Figure 3C). N_SC0_Agrp/Npy/Sst/Lrp1b (subsequently referred to simply as N_SC0) showed high expression of *Sst* and modest expression of *Agrp*, *Npy*, and *Lrp1b*. N_SC2_Agrp/Npy/Acvr1c (N_SC2) and N_SC9_Agrp/Npy (N_SC9) both showed high expression of *Agrp* and *Npy* and did not express *Lrp1b* but were differentiated by greater expression of *Acvr1c* in N_SC2.

Each of these subclusters was further analyzed using gene set enrichment to better describe the AgRP neuronal subtype identities (Supplementary Table 9). First, we examined the N_SC0 subcluster. Gene enrichment analysis of DEGs enriched in N_SC0 (114 genes versus N_SC2, 135 genes versus N_SC9) found the terms *Synaptic signaling* (GOBP, N_SC2 FE = 5.9, N_SC9 FE = 5.5), *cAMP signaling pathway* (KEGG, N_SC2 FE = 12.3, N_SC9 FE = 8.1), *G alpha i signaling events* (Reactome, N_SC2 FE = 6.3), and *AT_1_R pathway* (Biocarta, N_SC2 FE = 18.5) (Figure 3D and Supplementary Tables 10, 11). Second, we examined the N_SC2 subcluster. Gene enrichment analysis of DEGs enriched in N_SC2 (40 genes versus N_SC0, 83 genes versus N_SC9) found terms including *Adult feeding behavior* (GOBP, N_SC0 FE = 180.1), *Tandem pore domain potassium channels* (Reactome, N_SC0 FE = 147.3, N_SC9 FE = 53.8), and *Adipocytokine signaling pathway* (KEGG, N_SC0 FE = 34.2) (Figure 3D and Supplementary Tables 12, 13). Third, we examined the N_SC9 subcluster. Gene enrichment analysis of DEGs enriched in N_SC9 (54 genes versus N_SC0, 60 genes versus N_SC2) identified many terms that referred to the ribosome and its functions, including *Ribosome* (KEGG, N_SC0 FE = 108.0, N_SC2 FE = 66.8), *Large ribosomal subunit rRNA binding* (GOMF, N_SC0 FE = 154.8, N_SC2 FE = 94.3), and *SRP-dependent cotranslational protein targeting to membrane* (Reactome, N_SC0 FE = 141.9, N_SC2 FE = 82.5) (Figure 3D and Supplementary Tables 14, 15).

### Effects of DOCA-salt upon AgRP neurons

**FIGURE 4:**
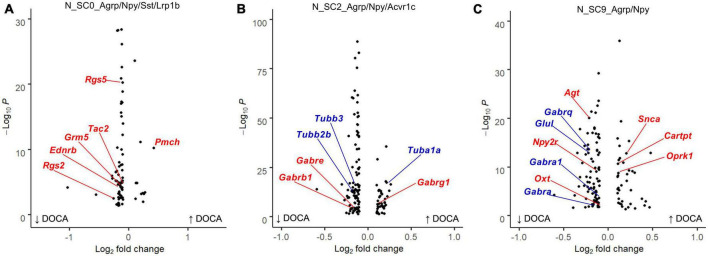
Deoxycorticosterone acetate (DOCA)-salt treatment differentially alters transcriptomes within Agouti-related peptide (AgRP) neuronal subtypes. **(A)** Differentially expressed genes between DOCA-salt and Sham conditions in N_SC0_Agrp/Npy/Sst/Lrp1b. Genes for the Reactome enrichment term *G alpha q signaling events* shown in red. **(B)** Differentially expressed genes between DOCA-salt and Sham conditions in N_SC2_Agrp/Npy/Acvr1c. Genes for GO Molecular Function enrichment term *GABA-gated chloride ion channel activity* shown in red, and for the Reactome term *Microtubule-dependent trafficking of connexons from Golgi to the plasma membrane* in blue. **(C)** Differentially expressed genes between DOCA-salt and Sham conditions in N_SC9_Agrp/Npy. Genes for GO Biological Process term *Regulation of catecholamine secretion* shown in red, and for KEGG term *GABAergic synapse* in blue.

Next, we examined the response of each AgRP subcluster to DOCA-salt treatment (Supplementary Table 16). In response to DOCA-salt, N_SC0 nuclei exhibited 80 dysregulated genes (11 upregulated, 69 downregulated) compared to Sham (Figure 4A). Terms related to GPCR signaling were enriched in the DOCA-salt DEG list including *G protein-coupled receptor binding* (GOMF, FE = 6.0), *G alpha q signaling events* (Reactome, FE = 7.5), and *Neuroactive ligand-receptor interaction* (KEGG, FE = 6.3) (Supplementary Table 17).

Deoxycorticosterone acetate-salt caused altered expression of 135 genes in N_SC2 (41 upregulated, 94 downregulated) compared to Sham (Figure 4B). Many terms related to GABA signaling and synaptic signaling were enriched in the DOCA-salt DEG list including *Anterograde trans-synaptic signaling* (GOBP, FE = 5.9), *GABA-gated chloride ion channel activity* (GOMF, FE = 38.0), and *GABAergic synapse* (KEGG, FE = 11.1). Terms including *Semaphorin receptor binding* (GOMF, FE = 22.4) and *Microtubule-dependent trafficking of connexons from Golgi to the plasma membrane* (Reactome, FE = 30.9) were also enriched in N_SC2 DOCA-salt DEGs (Supplementary Table 18).

Finally, DOCA-salt caused altered expression of 122 genes in N_SC9 (40 upregulated, 82 downregulated) compared to Sham (Figure 4C), including *Regulation of catecholamine secretion* (GOBP, FE = 17.9), *Melanocortin receptor binding* (GOMF, FE = 73.6), and *GABAergic synapse* (KEGG, FE = 8.3) (Supplementary Table 19).

### Effects of DOCA-salt upon microglia

**FIGURE 5:**
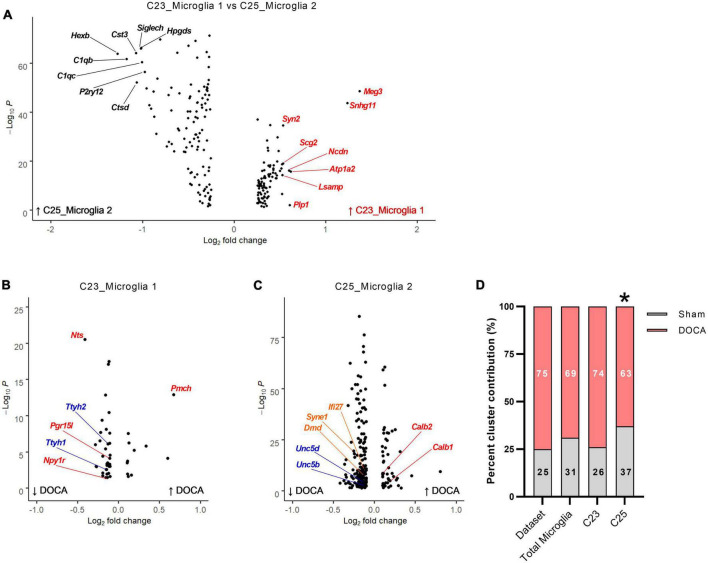
Deoxycorticosterone acetate (DOCA)-salt treatment alters transcriptomes of both resting and active microglia. **(A)** Differentially expressed genes between C23 and C25. The top eight genes enriched in C23 are shown in red and in C25 are shown in black. **(B)** Differentially expressed genes between DOCA-salt and Sham conditions in C23. Genes for Gene Ontology Biological Process (GOBP) term *Neuropeptide signaling pathway* are shown in red and for GO Molecular Function term *Volume-sensitive chloride channel activity* are shown in blue. **(C)** Differentially expressed genes between DOCA-salt and Sham conditions in C25. Genes for GO Molecular Function terms *Calcium ion binding involved in regulation of cytosolic calcium ion concentration*, *Netrin receptor activity*, and *Lamin binding* are shown in red, blue, and orange, respectively. **(D)** Percent DOCA-salt and Sham contributions of nuclei to the entire dataset, to both microglia clusters, to C23_Microglia 1, and to C25_Microglia 2. Deviation from dataset percentages determined by Chi-square test, *p < 0.05.

A role for microglia in the cerebrovascular effects of DOCA-salt treatment has been previously identified (Koizumi et al., 2019), prompting additional exploration of the effect of DOCA-salt upon microglia in the current dataset. Two distinct clusters (C23 and C25) were identified as microglia (Figure 2). Comparison of gene enrichment analysis patterns (Figure 5A and Supplementary Table 20) supported the conclusion that C23_Microglia 1 represents “resting” microglia and C25_Microglia 2 represents “activated” microglia. Gene enrichment analysis of DEGs enriched in C23 (“resting” microglia) found terms such as *Regulation of microtubule polymerization* (GOBP, FE = 22.6), *Cytoskeletal regulatory protein binding* (GOMF, FE = 171.6), and *GABA receptor binding* (GOMF, FE = 32.2) (Supplementary Table 21). These nuclei had 46 DOCA-salt dysregulated genes (11 upregulated, 35 downregulated) compared to Sham (Figure 5B and Supplementary Table 22). Terms with the greatest fold enrichment were *Neuropeptide signaling pathway* (GOBP, FE = 19.1) and *Volume-sensitive chloride channel activity* (GOMF, FE = 248.7) (Supplementary Table 23).

Gene enrichment analysis of DEGs enriched in C25 (“active” microglia) found terms such as *Phagocytosis* (GOBP, FE = 12.6), *Adaptive immune response* (GOBP, FE = 9.7), and *Interleukin-6 receptor binding* (GOMF, FE = 55.3) (Supplementary Table 24). These nuclei had 214 DOCA-salt dysregulated genes (45 upregulated, 169 downregulated) compared to sham, the most DOCA-salt DEGs in this dataset (Figure 5C and Supplementary Table 22). In the DOCA-salt DEGs, the GOBP terms *Regulation of synaptic plasticity* (FE = 8.4) and *Locomotion* (FE = 2.7) were enriched (Supplementary Table 25). GOMP terms for *Calcium ion binding involved in regulation of cytosolic calcium ion concentration* (FE = 105.7), *Netrin receptor activity* (FE = 35.2), and *Lamin binding* (FE = 18.7) were also enriched in DOCA-salt DEGs.

To determine whether DOCA-salt treatment changes microglia activation status within the ARC, we compared the percent of microglia in each snRNAseq microglia cluster (i.e., C23_Microglia 1 vs. C25_Microglia 2) to the dataset nuclei percentages between DOCA-salt treatment conditions (Figure 5D). Percent of microglia in both C23 and C25 in Sham and DOCA-salt samples did not deviate from the dataset percent (χ^2^ *p* = 0.1659), again consistent with no change in the total number of microglia in the DOCA-salt ARC. Examining clusters individually, we found that while the percent of microglia in Sham and DOCA-salt in C23 did not deviate from the dataset percent (χ^2^ *p* = 0.8176), the percent of microglia in DOCA-salt in C25 did significantly deviate from Sham (χ^2^ *p* = 0.0056). These results support the unexpected conclusion that there may be fewer activated microglia in the ARC during DOCA-salt treatment.

**FIGURE 6:**
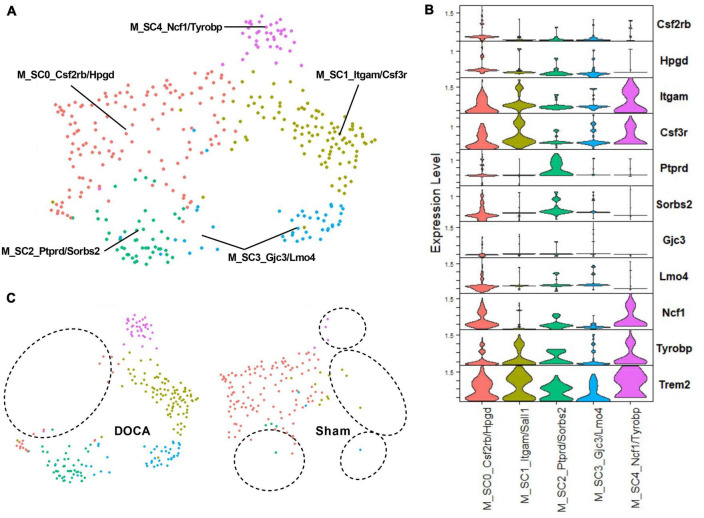
Deoxycorticosterone acetate (DOCA)-salt treatment appears to shift subtypes of activated microglia. **(A)** Uniform manifold approximation and projection (UMAP) plot of nuclei subclusters identified by Seurat within C25_Microglia 2. **(B)** Expression of the top two marker genes that uniquely identify subclusters within C25. **(C)** UMAP plot of nuclei clusters, split by condition (Sham, DOCA-salt). Circles highlight the absence of subclusters within each condition.

To further explore the effect of DOCA-salt upon activated microglia in the ARC, C25_Microglia 2 was subclustered to assess whether function(s) or abundances of activated microglial subtypes change in with DOCA-salt. Five subclusters of activated microglia were identified (Figures 6A, B and Supplementary Table 7). Interestingly, Sham C25 generated the bulk of M_SC0_Csf2rb/Hpgd while DOCA-salt C25 was the dominant contributor to the four other subclusters of activated microglia (M_SC1_Itgam/Sall1, M_SC2_Ptprd/Sorbs2, M_SC3_Gjc3/Lmo4, M_SC4_Ncf1/Tyrobp) (Figure 6C and Supplementary Table 26). While *Trem2* was a common marker gene for activated microglia subclusters, the highest *Trem2* expression value was observed in M_SC4_Ncf1/Tyrobp (Supplementary Table 26). These results support the conclusion that there is a difference in microglial activation state after DOCA-salt treatment.

## Discussion

The local RAS within the ARC is implicated in the integrated control of energy expenditure and may also contribute to blood pressure control (Claflin et al., 2017; Shi et al., 2017, 2021, 2022; Morselli et al., 2018). Previous work demonstrates that DOCA-salt stimulates both energy expenditure and blood pressure through mechanisms that involve the brain RAS, and increasing evidence supports a major role for the ARC RAS in the integrated control of energy expenditure in response to various stimuli through the actions of AT_1_R within selected AgRP neurons (Itaya et al., 1986; Kubo et al., 2000; Park and Leenen, 2001; Grobe et al., 2011; Claflin et al., 2017; Morselli et al., 2018; Oliveira et al., 2022). The current study builds upon these insights by reporting high resolution profiles of ARC cell type and subtype-specific responses to DOCA-salt treatment. Our data further reveals that DOCA-salt caused unique effects within novel subtypes of ARC AgRP neurons and alters the activation status of microglia within the ARC.

Previous work supports the hypothesis that there are multiple distinct subtypes of AgRP neurons within the ARC, but unique molecular markers identifying these subtypes and the physiological roles of each subtype remain unclear. We identified three distinct subtypes, primarily characterized by co-expression of *Sst*, *Acvr1c*, or neither. Based on the previous co-localization of *Agtr1a* and *Sst* in a specific subset of AgRP neurons by Romanov et al. and our implication of AT_1_R signaling within AgRP neurons in the control of energy expenditure (Claflin et al., 2017; Romanov et al., 2017; Sapouckey et al., 2017; Morselli et al., 2018), we hypothesize that this cluster (N_SC0) represents the AT_1_R-expressing subset of AgRP neurons that is critical for the integrated control of energy expenditure in response to various stimuli such as DOCA-salt or leptin (Claflin et al., 2017; Morselli et al., 2018). While AgRP clusters marked with *Sst* and *Acvr1c* have been reported, this is the first report of a third AgRP subtype in N_SC9. Beyond *Agrp* and *Npy*, markers genes for N_SC9 were lncRNAs with relatively low expression, and therefore additional study of this subtype will be difficult.

Microglia are implicated in cerebrovascular control during DOCA-salt treatment (Koizumi et al., 2019). The current study builds upon that observation by reporting that DOCA-salt causes changes in the subtype of activated microglia without robust effects on total proportions of resting and active microglia. Further understanding the specific effects of DOCA-salt-induced active type switching, however, is hampered by limited understanding of the pathophysiological roles of individual activated subtypes. Nonetheless, we can propose identities based on activated microglia subtype marker genes. In M_SC1, increased expression of *Itgam*/Cd11b is a well-known marker of activated microglia (Holtman et al., 2017; Jurga et al., 2020). *Ptprd*/PTPσ plays an inhibitory role for phagocytosis in neurotoxic microglia (Dyck et al., 2018) and other marker genes *Gria2* and *Gria3* encode group II metabotropic glutamate receptors that regulate microglial transformation into the neurotoxic phenotype (Domercq et al., 2013), suggesting that M_SC2 is a neurotoxic subtype. In M_SC3, *Gjc3* expression has been shown to increase in microglia associated with gliomas and near trauma sites (Szulzewsky, 2015), and transcriptional regulator *Lmo4* is a coactivator in the *TGF*β signaling pathway (Lu et al., 2006), a critical pathway the regulates microglia adaptation (Zöller et al., 2018). Finally in M_SC4, increased expression of *Ncf1*/p47phox has been reported in phagocytic microglia (Haslund-Vinding et al., 2017) and upregulation of *Tyrobp* and *Trem2* (a M_SC4 top marker gene) are known markers for DAMs, Alzheimer’s disease-associated microglia with phagocytic activity (Keren-Shaul et al., 2017).

In resting microglia, DOCA-salt downregulated genes involved in volume-sensitive chloride channel activity, which may hinder microglia membrane depolarization and ramification (Eder, 1998). DOCA-salt also dysregulated neuropeptide signaling pathway genes in resting microglia likely impacting general functions of this cell type (Geloso et al., 2015). In the primary cluster of activated microglia, DOCA-salt-induced transcriptomic changes showed upregulation of calcium signaling genes and downregulation of netrin receptor and lamin binding genes. These expression changes may result in altered ability of microglia to depolarize membranes and respond to neuronal activity (Eder, 1998; Umpierre et al., 2020), may prevent DOCA-salt activated microglia from interacting with surrounding neurons (Fujita et al., 2020), and may indicate that activated microglia have reduced ability to regulate gene expression (Pascual-Reguant et al., 2018). Whether these effects are specific to DOCA-salt-induced activated microglia subtypes is yet to be determined. Thus, as the roles of these individual active microglia subtypes are clarified in future studies, the significance of their promotion by DOCA-salt should become clear.

Single-cell or -nucleus RNAseq approaches have important limitations when it comes to highly dimensional datasets (Kharchenko, 2021). Genes with high cell type specificity, such as RAS components, are lowly expressed on average in a dataset containing many cell types and are often lost in the normalization procedures. Many experiments that support an important role for the RAS within the brain in various physiological endpoints are interventional studies that utilized genetic and/or pharmacological methods to establish causal relationships and functional roles for the RAS within the brain. For example, we have demonstrated that genetic deletion of AT_1A_ from AgRP neurons has profound effects on metabolism but not blood pressure (Claflin et al., 2017). Future studies utilizing more targeted methods are also required to dissociate the primary effects of DOCA itself, or sodium loading versus the complex secondary effects of this combined treatment upon gene expression patterns.

This study had some limitations. Rodents generally exhibit sexual dimorphism with regard to cardiovascular responses to DOCA-salt, with females exhibiting less extreme responses than males (Belanger et al., 2020). Here we studied males to capture the more extreme DOCA-salt response. There are also many other brain regions that participate in the brain RAS and may respond to DOCA-salt treatment. Our recent publications direct us toward examining the ARC because interferences with AT1A in the ARC or melanocortin type 4 receptor signaling abolishes the metabolic stimulatory effects of DOCA-salt (Claflin et al., 2017; Morselli et al., 2018; Oliveira et al., 2022). In contrast, disrupting AT1A in the SFO or SON does not interrupt the metabolic stimulatory effects of DOCA-salt (Hilzendeger et al., 2013; Sandgren et al., 2018). Future studies are needed to compare the ARC cell-specific transcriptomes in females after DOCA-salt treatment and to investigate effects of DOCA-salt in other brain regions. Many imaging methods have been developed and compared for assessing microglial activation (Eme-Scolan and Dando, 2020), and these methods are the most common approach to identifying activated microglia. Emerging methods, such as snRNAseq, complement and extend those approaches through the ability to distinguish subtypes of microglia based on their transcriptome rather than morphology. Additional studies must be done to better understand the impact of DOCA-salt treatment upon microglial activation status.

In conclusion, DOCA-salt is a model of low-renin hypertension and hypermetabolism, and this model is associated with and dependent upon induction of the brain RAS and activation of the angiotensin AT_1_R within specific brain regions and cell types. Using snRNAseq methods, here we established the transcriptomic effects of DOCA-salt within individual cell types of the ARC, thereby providing a novel toolbox for investigators to explore cell-specific effects within this model. We have identified multiple distinct subtypes of AgRP neurons within the ARC and established the unique transcriptomic effects of DOCA-salt within these individual neuronal subtypes. Finally, our results highlight specific effects of DOCA-salt to alter the activation status but not abundance of microglia within the ARC. These findings, and the associated sequencing dataset, provide exciting new insight into cell-specific effects of DOCA-salt within the brain, which ultimately informs our understanding of the hypothalamic mediators and consequences of low-renin hypertension.

## Data availability statement

The datasets presented in this study can be found in online repositories. The names of the repository/repositories and accession number(s) can be found below: https://www.ncbi.nlm.nih.gov/geo/, GSE221367.

## Ethics statement

This animal study was reviewed and approved by the University of Iowa and Medical College of Wisconsin Institutional Animal Care and Use Committees.

## Author contributions

VW: snRNAseq data analysis, data interpretation, and manuscript preparation. GD: DOCA-salt animal experiment, nuclei isolation, and sequencing. HC: experimental design. PN and CS: data interpretation and manuscript preparation. KC, MR, and LM: data collection from DOCA-salt animals. JG: supervision of animal and laboratory-based experiments, experiment design, data analysis, data interpretation, and manuscript preparation. AK: supervision of data analysis, data interpretation, and manuscript preparation. All authors contributed to the article and approved the submitted version.
