# Supplementary material for: Cell-specific transcriptome changes in the hypothalamic arcuate nucleus in a mouse deoxycorticosterone acetate-salt model of hypertension
Source: Front Cell Neurosci. 2023 May 24;17:1207350. doi: 10.3389/fncel.2023.1207350 (PMC10244568; doi:10.3389/fncel.2023.1207350)
Supplement: Supplementary file 21 [file Data_Sheet_21.DOCX]

**ONLINE SUPPLEMENT**

**Cell-type specific transcriptome changes in the hypothalamic arcuate nucleus in a mouse deoxycorticosterone acetate (DOCA)-salt model of hypertension**

Valerie A. Wagner^1,2^, Guorui Deng^3^, Kristin E. Claflin^3^, McKenzie L. Ritter^1^, Huxing Cui^3,4,5^, Pablo Nakagawa^1,6^, Curt D. Sigmund^1,6,7^, Lisa L. Morselli^8^, Justin L. Grobe^1,6,7,9,10,*^, and Anne E. Kwitek^1,6,10,11,*^

^1^Department of Physiology, Medical College of Wisconsin, Milwaukee, WI

^2^Genetics Graduate Program, University of Iowa, Iowa City, IA

^3^Department of Neuroscience and Pharmacology, University of Iowa, Iowa City, IA

^4^Obesity Research and Education Initiative, University of Iowa, Iowa City, IA

^5^Fraternal Order of Eagles Diabetes Research Center, University of Iowa, Iowa City, IA

^6^Cardiovascular Center, Medical College of Wisconsin, Milwaukee, WI

^7^Neuroscience Research Center, Medical College of Wisconsin, Milwaukee, WI

^8^Department of Medicine, Division of Endocrinology and Molecular Medicine, Medical College of Wisconsin, Milwaukee, WI

^9^Comprehensive Rodent Metabolic Phenotyping Core, Medical College of Wisconsin, Milwaukee, WI

^10^Department of Biomedical Engineering, Medical College of Wisconsin, Milwaukee, WI

^11^Linda T. and John A. Mellowes Center for Genomic Sciences and Precision Medicine, Medical College of Wisconsin, Milwaukee, WI

* denotes co-corresponding authors

Running title: snRNAseq of arcuate nucleus after DOCA-salt

Correspondence:

Justin L. Grobe, PhD

Department of Physiology

Medical College of Wisconsin

8701 Watertown Plank Rd

Milwaukee, WI 53226

[jgrobe@mcw.edu](mailto:jgrobe@mcw.edu)

(414) 955-4981

or

Anne E. Kwitek, PhD

Department of Physiology

Medical College of Wisconsin

8701 Watertown Plank Rd

Milwaukee, WI 53226

[akwitek@mcw.edu](mailto:akwitek@mcw.edu)

(414) 955-8241

**Table S1. Sample Cohort**

| Condition | Animal ID | ARC Punch Count | Sample ID (samples pooled after nuclei isolation) |
| --- | --- | --- | --- |
| Sham | 539_4 | 2 | Sham 1 |
|  | 540_1 | 2 |  |
|  | 541_2 | 2 | Sham 2 |
|  | 540_3 | 1 |  |
| DOCA | 539_1 | 2 | DOCA 1 |
|  | 539_3 | 2 |  |
|  | 539_5 | 1 | DOCA 2 |
|  | 540_2 | 2 |  |

Bilateral arcuate nucleus (ARC) punches (10µm thick, 0.74 diameter) were obtained from brains of Sham (n=4) and DOCA-salt treated (n=4) mice, and nuclei were isolated. Nuclei obtained from individual mice were then quantitated and pooled to generate four pooled samples for snRNAseq with roughly similar abundances.

**Table S2.** **Quality control metric comparison of raw datasets versus a previously published single nucleus RNA-seq study of the mouse arcuate nucleus of the hypothalamus.**

|  | ***Samples from current study*** | | | | ***Samples from our previous study (PMC7347451)*** | | | | | |
| --- | --- | --- | --- | --- | --- | --- | --- | --- | --- | --- |
| **Sample / Mouse_ID:** | **Sham 1:**  **539-4 &**  **540-1** | **Sham 2:**  **541-2 &**  **540-3** | **DOCA 1:**  **539-1 &**  **539-3** | **DOCA 2:**  **539-5 &**  **540-2** | **536-1** | **536-3** | **536-5** | **536-2 &**  **536-4** | **537-1 &**  **537-3** | **537-5 &**  **538-2** |
| **Metrics before application of exclusion criteria** | | | | | | | | | | |
| **Estimated # of Cells** | 2,805 | 8,325 | 10,765 | 19,754 | 5,436 | 4,083 | 1,871 | 7,087 | 2,842 | 6,238 |
| **Median Genes per Cell** | 4,482 | 2,156 | 1,638 | 1,684 | 2,617 | 2,227 | 2,357 | 2,309 | 3,391 | 1,125 |
| **Reads Mapped Confidently to Genome (%)** | 89.2 | 87.6 | 91.0 | 92.0 | 78.7 | 87.1 | 87.6 | 83.2 | 86.8 | 85.2 |
| **Reads Mapped Confidently to Intergenic Regions (%)** | 6.8 | 7.2 | 7.0 | 6.5 | 6.0 | 6.4 | 6.8 | 6.1 | 7.2 | 6.5 |
| **Reads Mapped Confidently to Intronic Regions (%)** | 35.7 | 36.7 | 41.3 | 35.1 | 33.3 | 36 | 35.2 | 37.1 | 39.5 | 30.8 |
| **Reads Mapped Confidently to Exonic Regions (%)** | 46.8 | 43.8 | 42.7 | 50.4 | 39.4 | 44.7 | 45.5 | 40 | 40.1 | 47.8 |
| **Reads Mapped Confidently to Transcriptome (%)** | 43.9 | 41.1 | 39.8 | 47.7 | 37.3 | 42.6 | 43.2 | 37.9 | 37.9 | 45.7 |
| **Reads Mapped Antisense to Gene (%)** | 0.9 | 0.9 | 1.1 | 1.0 | 1.0 | 1.0 | 1.1 | 0.9 | 1.0 | 1.0 |
| **Total Genes Detected** | 21,084 | 21,557 | 21,253 | 21,584 | 21,399 | 21,686 | 20,700 | 21,762 | 21,395 | 21,316 |
| **Mean Reads per Cell** | 92,993 | 34,284 | 23,438 | 13,504 | 42,009 | 70,922 | 169,944 | 43,891 | 114,980 | 48,545 |
| **Sequencing Saturation (%)** | 34.9 | 27.2 | 29.4 | 20.0 | 30.7 | 45.6 | 75.7 | 36.9 | 59.1 | 49.1 |
| **Total Nuclei detected** | 2,203 | 7,759 | 10,307 | 18,906 | 4,996 | 16 | 1,609 | 6,421 | 2,405 | 5,570 |

**Table S3. Tissue masses after three weeks of DOCA-salt treatment.**

| **Tissue** | **Sham (n=7)** | **DOCA-salt (n=8)** | **p-value** |
| --- | --- | --- | --- |
| Interscapular brown adipose (mg)  (mg/g) | 75.7±11.8  4.9±0.4 | 105.7±14.7  4.1±0.5 | 0.140  0.103 |
| Inguinal white adipose (mg)  (mg/g) | 309±27  11.5±1.0 | 397±51  15.3±1.7 | 0.340  0.218 |
| Peri-gonadal white adipose (mg)  (mg/g) | 336±57  12.9±2.3 | 486±54  18.7±1.7 | 0.068  0.051 |
| Heart ventricles (mg)  (mg/g) | 138±8  5.3±0.4 | 124±5  4.8±0.1 | 0.142  0.241 |
| Liver (g)  (mg/g) | 1.14±0.05  43.8±2.7 | 1.31±0.06  51.2±2.4 | 0.066  0.053 |
| Kidneys (mg)  (mg/g) | 346±14  13.3±0.7 | 427±11  16.7±0.3 | 0.002  <0.001 |
| Adrenal glands (mg)  (mg/g) | 4.9±0.4  0.19±0.02 | 2.9±0.33  0.12±0.02 | 0.006  0.804 |

Data presented as mean±sem, and analyzed using 2-tailed independent t-tests.

**Table S5. Count and percent of Sham and DOCA-salt samples contributing to each cluster.**

| Cluster | Sham | | DOCA-salt | |
| --- | --- | --- | --- | --- |
|  | Nuclei Count | Nuclei Percent (%) | Nuclei Count | Nuclei Percent (%) |
| C0_Snhgll/Meg3 | 807 | 20.7 | 3090 | 79.3 |
| C1_Gm42418/Rpl41 | 1251 | 40 | 1880 | 60 |
| C2_GABAergic | 448 | 14.5 | 2650 | 85.5 |
| C3_Gm42418/Rps12 | 973 | 32.9 | 1985 | 67.1 |
| C4_Astrocytes 1 | 793 | 27.4 | 2100 | 72.6 |
| C5_Glutamatergic | 561 | 22.3 | 1952 | 77.7 |
| C6_Nap1i5/Isl1 | 779 | 31 | 1732 | 69 |
| C7_Astrocytes 2 | 370 | 20.2 | 1463 | 79.8 |
| C8_Oligodendrocytes 1 | 548 | 34 | 1064 | 66 |
| C9_Sim1 | 283 | 20.5 | 1095 | 79.5 |
| C10_Oligodendrocytes 2 | 324 | 26.9 | 881 | 73.1 |
| C11_preOligodendrocyte | 299 | 25.1 | 892 | 74.9 |
| C12_Pomc | 441 | 38.9 | 692 | 61.1 |
| C13_Endothelium | 276 | 29.6 | 655 | 70.4 |
| C14_Rgs16 | 161 | 17.4 | 762 | 82.6 |
| C15_Arpp21/Meis2 | 4 | 0.4 | 910 | 99.6 |
| C16_Sst/Dlk1 | 228 | 25.7 | 658 | 74.3 |
| C17_Ghrh | 179 | 23.7 | 575 | 76.3 |
| C18_Fezf1/Cacna2d1 | 102 | 13.7 | 645 | 86.3 |
| C19_Agrp | 246 | 35 | 456 | 65 |
| C20_Tac1/Ar | 64 | 12.3 | 458 | 87.7 |
| C21_Tanycytes | 143 | 28 | 367 | 72 |
| C22_KNDy | 148 | 32.7 | 305 | 67.3 |
| C23_Microglia 1 | 119 | 26.3 | 334 | 73.7 |
| C24_Tac1/Rreb1 | 2 | 0.5 | 374 | 99.5 |
| C25_Microglia 2 | 140 | 37.3 | 235 | 62.7 |
| C26_Pmch | 14 | 4 | 339 | 96 |
| C27_Astrocytes 3 | 78 | 24.8 | 236 | 75.2 |
| C28_Ptgds/Atp1a2 | 83 | 36.1 | 147 | 63.9 |
| C29_Slc18a2/Hdc | 59 | 29.5 | 141 | 70.5 |
| C30_Slc5a7/Elavl2 | 10 | 10.8 | 83 | 89.2 |
| C31_Oligodendrocytes 3 | 29 | 33.7 | 57 | 66.3 |

**Table S8. Count and percent of Sham and DOCA-salt samples contributing to each neuropeptide-related subcluster.**

| Cluster | Sham | | DOCA-salt | |
| --- | --- | --- | --- | --- |
|  | Nuclei Count | Nuclei Percent (%) | Nuclei Count | Nuclei Percent (%) |
| N_SC0_Agrp/Npy/Sst/Lrp1b | 203 | 33.1 | 411 | 66.9 |
| N_SC1_Lamp5/Tcf7l2 | 218 | 35.7 | 393 | 64.3 |
| N_SC2_Agrp/Npy/Acvr1c | 151 | 28.6 | 377 | 71.4 |
| N_SC3_Six3/Col25a1 | 107 | 25.5 | 312 | 74.5 |
| N_SC4_KNDy | 128 | 31.5 | 278 | 68.5 |
| N_SC5_Ghrh/Gal | 82 | 26.0 | 233 | 74.0 |
| N_SC6_Crabp1/Vgll3 | 113 | 38.8 | 178 | 61.2 |
| N_SC7_Cartpt/Trh | 43 | 16.3 | 220 | 83.7 |
| N_SC8_Pomc/Cartpt | 96 | 50.3 | 95 | 49.7 |
| N_SC9_Agrp/Npy | 69 | 46.6 | 79 | 53.4 |
| N_SC10_Gal/Gpr50 | 6 | 8.3 | 66 | 91.7 |
| N_SC11_Slc6a3/Th | 26 | 37.1 | 44 | 62.9 |

**Table S26. Count and percent of Sham and DOCA-salt samples contributing to each cluster of activated microglia subclusters.**

| Cluster | Sham | | DOCA-salt | |
| --- | --- | --- | --- | --- |
|  | Nuclei Count | Nuclei Percent (%) | Nuclei Count | Nuclei Percent (%) |
| M_SC0_Csf2rb/Hpgd | 118 | 86.1 | 19 | 13.9 |
| M_SC1_Itgam/Sall1 | 11 | 10.5 | 94 | 89.5 |
| M_SC2_Ptprd/Sorbs2 | 4 | 8.2 | 45 | 91.8 |
| M_SC3_Gjc3/Lmo4 | 3 | 6.8 | 41 | 93.2 |
| M_SC4_Ncf1/Tyrobp | 4 | 10.0 | 36 | 90.0 |


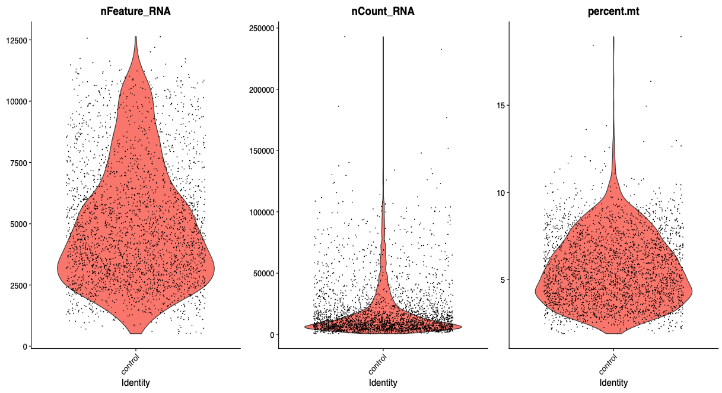


**Sham 1 (539-4 & 540-1)**


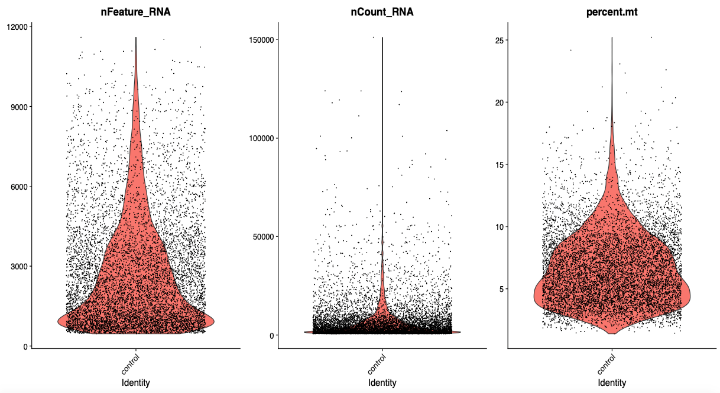


**Sham 2 (541-2 & 540-3)**


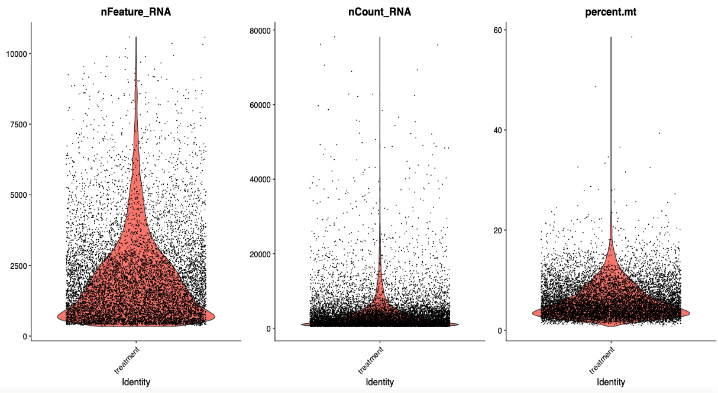


**DOCA 1 (539-1 & 539-3)**


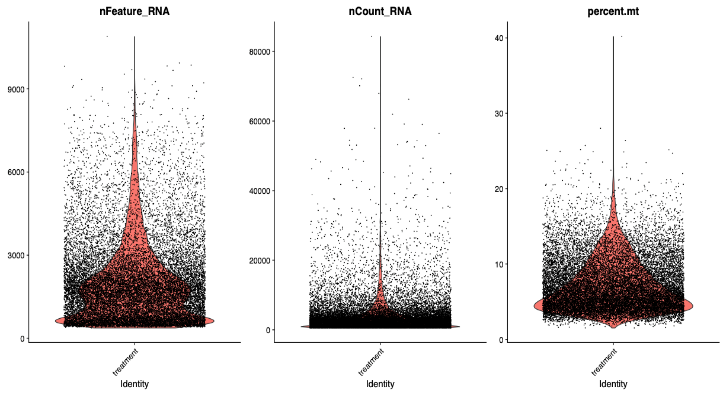


**DOCA 2 (539-5 & 540-2)**

**A**

**B**

**C**

**D**

**Figure S1.** **Quality control metrics prior to application of exclusion criteria in arcuate nucleus of the hypothalamus isolated nuclei.** Expressed gene count (nFeature), transcript count (nCount_RNA), and percent mitochondrial gene expression (percent.mt) from Sham (A) and DOCA-salt treated datasets (B). Percent of nuclei contributing to study dataset split by experimental conditions (Sham, DOCA) (C) and by sample ID (D).

**Sham 1(539-4 & 540-1)**

**Sham 2 (541-2 & 540-3)**

**DOCA 1 (539-1 & 539-3)**

**DOCA 2 (539-5 & 540-2)**

**A**

**B.**

**C**

**D**


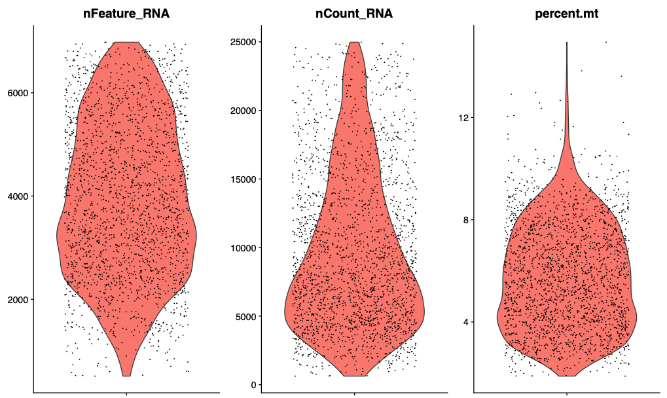

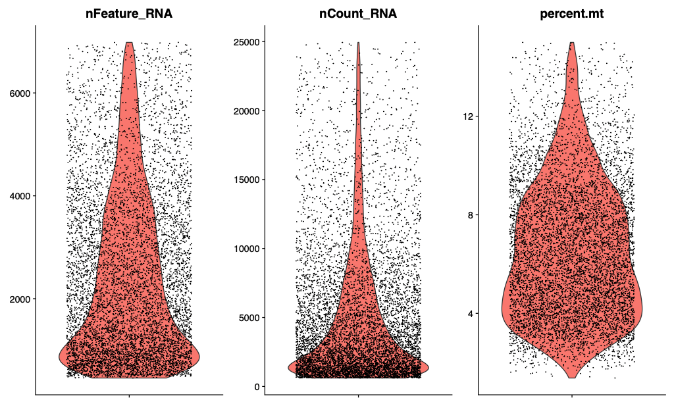

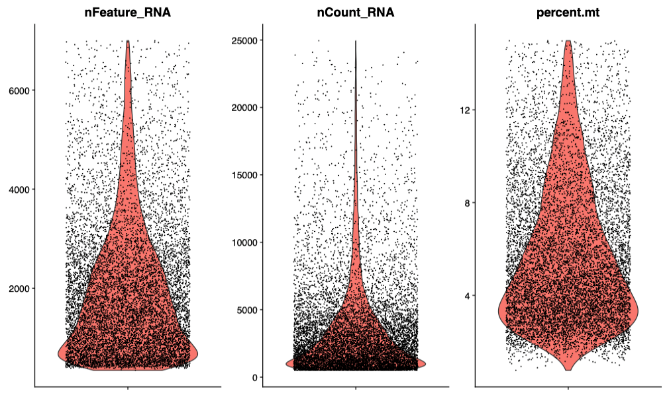

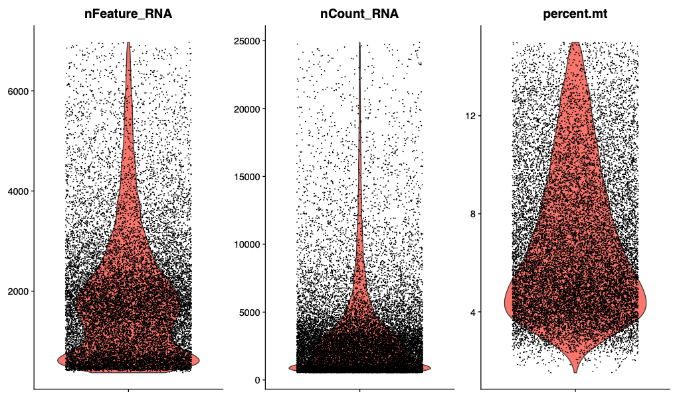


**Figure S2.** **Quality control metrics after application of exclusion criteria in arcuate nucleus of the hypothalamus isolated nuclei.** Expressed gene count (nFeature), transcript count (nCount_RNA), and percent mitochondrial gene expression (percent.mt) from Sham (A) and DOCA-salt treated datasets (B). Percent of nuclei contributing to study dataset split by experimental conditions (Sham, DOCA) (C) and by sample ID (D).
